# Persistent *H. pylori *colonization in early acquisition age of mice related with higher gastric sialylated Lewis x, IL-10, but lower interferon-γ expressions

**DOI:** 10.1186/1423-0127-16-34

**Published:** 2008-12-27

**Authors:** Yao-Jong Yang, Hsiao-Bai Yang, Jiunn-Jong Wu, Bor-Shyang Sheu

**Affiliations:** 1 Departments of Pediatrics, Medical College, National Cheng Kung University, Taiwan, Republic of China; 2 Department of Pathology, Medical College, National Cheng Kung University, Taiwan, Republic of China; 3 Department of Medical Laboratory Science and Biotechnology, Medical College, National Cheng Kung University, Taiwan, Republic of China; 4 Department of Internal Medicine, Medical College, National Cheng Kung University, Taiwan, Republic of China; 5 Institute of Clinical Medicine, Medical College, National Cheng Kung University, Taiwan, Republic of China; 6 Department of Pathology, Ton-Yen General Hospital, Hsinchu County, Taiwan, Republic of China

## Abstract

**Background:**

*H. pylori *infection is less prevalent in childhood. This study validated whether the rates of *H. pylori *colonization depend on different acquisition ages, and correlate with the different gastric Lewis antigens or cytokine expressions after *H. pylori *acquisition.

**Methods:**

We applied a young (7-day-old) C57BL/6 mice group (n = 50) and adult (6-week-old) C57BL/6 mice group (n = 50). In each group, 30 mice were challenged with *H. pylori *and 20 mice served as naïve control. The success of *H. pylori *colonization was assessed on the 2^nd ^week and the 8^th ^week, respectively. The intensity of the Lewis x, sialylated Lewis x(sialyl-Le^x^), and cytokine expressions, including TNF-α, IFN-γ, IL-6, IL-10, and IL-1β, were immunochemically stained and graded.

**Results:**

On the 2^nd ^week after *H. pylori *challenge, the colonization rates of *H. pylori *were similar between the young mice group and the adult mice group (89% vs. 100%, *P *> 0.05). However, on the 8^th ^week, the *H. pylori *colonization rate was significantly lower in the young mice group than in the adult mice group (53% vs. 95%, *P *= 0.003). On the 8^th ^week, the young mice with a persistence of *H. pylori *colonization had higher sialyl-Le^x^, higher IL-10, and lower IFN-γ than those of the mice that lost colonization during the 2^nd ^to the 8^th ^week (*P *< 0.05).

**Conclusion:**

The persistence of *H. pylori *colonization could be an acquisition-age determinant process. After *H. pylori *exposure at an early acquisition age, the host response with a higher sialyl-Le^x ^and IL-10, but a lower IFN-γ correlates to the consequent persistence of *H. pylori *colonization.

## Introduction

*Helicobacter pylori *infection can cause chronic gastritis and even peptic ulcer disease in both children and adults [1,2]. In epidemiological studies, the seropositivity of *H. pylori *has been shown to be less in children than in adults [3,4]. A follow-up cohort study showed that the highest incidence of seroconversion occurs in early childhood and gradually decreases with age [3]. Several studies have suggested that an early acquisition of *H. pylori *infection in childhood may possibly lead to a subsequent loss of colonization [3-6]. However, the exact regulations within children to determine either loss or persistence of *H. pylori *colonization after early acquisition remains not well-known.

A blood group binding adhesin (BabA) of *H. pylori *selectively binds to the fucosylated Lewis antigen (Le^b^) of gastric cells mediating a heavy bacterial colonization [7-9]. The other sialic acid-binding adhesin (SabA) of *H. pylori *binds to the gastric sialylated Lewis x antigen (sialyl-Le^x^) alternatively assisting the persistent colonization after chronic inflammation [10], and especially becomes important when the Le^b ^antigen of the host is absent or weak [11]. Our recent study disclosed that *H. pylori*-infected children had a significant increase of sialyl-Le^x^, but not Le^b ^expression [12]. It is thus interesting to check whether the persistence of the early acquisition of *H. pylori *in childhood could be related with the variable host responses of gastric inflammation or of epithelial sialylation to adapt the SabA of *H. pylori*.

With adult C57BL/6 mice older than six weeks of age, the clinical *H. pylori *strains can successfully colonize most of the mice for more than six months [13,14]. The chronic *H. pylori *infection leads to diverse Th1 type predominant gastric inflammations with variable pro-inflammatory and Th1-dominant type cytokine responses among different hosts [15-18]. Accordingly, the current study aimed at first to check whether the persistence of *H. pylori *colonization could be an acquisition-age determinant process. In addition, this study attempted to answer whether the variable sialyl-Le^x ^and the inflammatory cytokine expressions after *H. pylori *challenge could determine the persistent colonization of *H. pylori *infection, especially in mice receiving the challenge of early acquisition.

## Materials and methods

### Mice groups and *H. pylori *isolates for challenge experiment

This study used 100 C57BL/6 mice (50 seven-day-old young mice and 50 six-week-old adult male mice), including 60 in the experimental group challenged with a type 1, SabA-positive *H. pylori *clinical isolate (HP 625) and 40 in naïve controls. The multiplications of the *H. pylori *isolates were applied with the same method as our previous mice model study to achieve a density of 0.8 × 10^8 ^CFU/ml. [19]. For the experimental group, a bacteria suspension 0.1 ml for young mice and 0.5 ml for the adult mice was orally inoculated for three consecutive days, respectively. Ten each of the young and adult mice of the control group were sacrificed at the 2^nd ^and the 8^th ^week. In the experimental mice, ten each of the young and adult mice at the 2^nd ^week and 20 each of the young and adult mice at the 8^th ^week after inoculation were sacrificed by intra-peritoneal injection of pentobarbital (150–200 mg/kg). The isolated stomachs were incised along the greater and lesser curvatures into two halves. One half was formalin-embedded for histology and Lewis antigen examinations. The other half was immediately placed in buffer, frozen on dry ice, and stored at -80°C for cytokine examination. *H. pylori *infection was proven by a rapid urea test and histological examination.

### Gastric histology of mice

Two investigators including one pathologist, unaware of the demographic data and colonization results, analyzed the gastric histology. Applying the method of our previous mice model [19], the histological parameters, including *H. pylori *density (range 0–4), acute inflammatory score (AIS, range 0–3), chronic inflammation score (CIS, range 0–3), atrophic change (AT, range 0–1), and intestinal metaplasia (IM, range 0–3) were evaluated topographically over the antrum, corpus and cardia, respectively. The grading system of *H. pylori *density used was as follows: 0, no bacteria detected; 1, a mild level of colonization and bacteria not detected in every gastric crypt; 2, a mild level of colonization with bacteria detected in the majority of crypts present; 3, moderate to heavy colonization in all crypts; and 4, severe colonization with all crypts densely packed with bacteria. The results presented are the mean value of colonization for each group. The total *H. pylori *density (THPD) was defined as the sum of the densities obtained from the antrum, corpus and cardia, and thus ranged from 0 to 12. The total acute (TAIS) and chronic (TCIS) inflammatory scores were also a sum of the score from three locations (range 0–9).

Bacterial infection of *H. pylori *was defined as the presence of any bacteria in the gastric sections, while mice were considered to have failed to be inoculated when no bacteria were seen in histology and negative for the CLO test.

### Immunohistochemical staining and scoring for gastric Lewis expression

**Figure 1:**
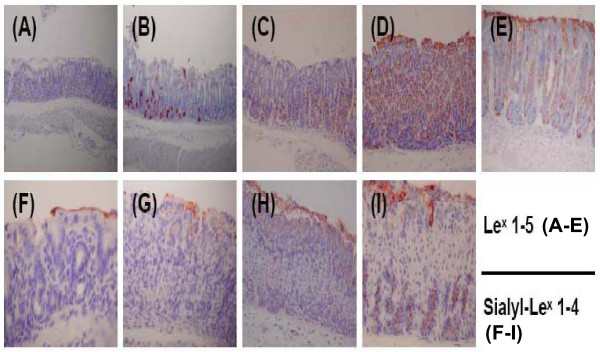
**The immunohistochemical (IHC) staining and grading of Lewis x (Le^x^), and sialyl-Lewis x (sialyl- Le^x^) antigens are shown for the gastric biopsies of mice (200×)**. The IHC staining showed negative for two Lewis antigens and was referred to as score 0. For Le^x^, score 1 (A): scant staining (< 5% in parietal cells of the deep glands; score 2 (B): deep glands 5–50% staining and weak; score 3 (C): deep glands > 50% staining and strong; score 4 (D): whole layer of deep glands with superficial epithelium staining, no mucus expression; score 5 (E): whole layer with mucus expression. For sialyl- Le^x^, score 1 (F): only surface mucus positive; score 2 (G): Mucus and upper epithelium; score 3 (H): upper epithelium and deep glands (chief cells < 50%); score 4 (I): upper epithelium and deep glands (chief cells ≥ 50%).

Immunostaining of gastric specimens for Lewis antigens was performed using the avidin-biotin-peroxidase technique with a VECTOR Ms.O.M. Immunodetection Kit. The primary monoclonal antibody (anti-Le^x^, Signet Laboratories, Inc., Dedham, MA) was selected to detect the gastric Le^x ^antigens [11,12]. Besides Le^x ^antigen staining, the monoclonal antibody against sialyl-Le^x ^(mouse IgM MAB2096, Chemicon International, Inc., Temecula, CA) was used [12]. Nonspecific binding sites were saturated with 2–2.5% bovine serum albumin. For each gastric site, the intensity of Le^x ^(range 0–5) and sialyl-Le^x ^(range 0–4) was scored according to the topography of the staining (Figure 1). Total gastric Lewis antigen intensity for Le^x ^and sialyl-Le^x ^was the sum of the antrum, corpus and cardia (range 0–15 in Le^x ^and 0–12 in sialyl-Le^x^).

### Immunohistochemical staining and scoring for cytokines expression

The frozen sections (4 μm) were dried at room temperature for 2 h, and fixed with 3.7% formaldehyde for 1 min. After washing with PBS twice, the samples were fixed with cold acetone and then washed with PBS. The Mouse Ig Blocking Reagent (VECTOR Laboratories, Burlingame, CA, U.S.A.) was used for blocking nonspecific binding sites. The rat anti-mouse monoclonal antibodies (TNF-α, IL-6, IL-10, IL-1β, and IFN-γ) and the rabbit anti-mouse polyclonal antibody (IL-1β) were used as the primary antibody. The HRP polymer conjugate broad spectrum (Zymed Laboratories Inc. South San Francisco, CA, U.S.A.) was used as the 2^nd ^antibody. The AEC kit was used to illustrate the stain. The intensity of each cytokine was scored according to the distribution of positive staining cells on the epithelium (range 0–3), and on the lamina propria components, including the matrix, polymorphonuclear cells, lymphocytes, and fibrocytes (Table 1).

**Table 1:** Scoring of cytokine expressions on the gastric epithelium and lamina propria.

	Score			
	
	0	1	2	3
**Epithelium**	Negative	Less than 33%	33%–67%	More than 67%
**Lamina propria**				
*Matrix*	Absence	Presence		
*Lymphocyte*	Absence	Presence		
*Fibrocyte*	Absence	Presence		
*Neutrophil*	Absence	Presence		

The quantitative scoring of each cytokine on the epithelium was as follows: score 1: positive staining in the cytoplasma of epithelial cells only on the upper 1/3 part of the epithelium; score 2: on the upper and middle part of the epithelium (1/3–2/3); score 3: on the whole layer of the epithelium (> 2/3). The positive staining of each cytokine on the lamina propria (lymphocytes, fibrocytes, and neutrophils) means there were more than five positive cells in a high power field of view in the microscopic examination.

### Statistical analysis

The Pearson's χ^2 ^test was used to test the difference of colonization rates between the experimental and control mice. The histological parameters, Lewis antigens, and the cytokine expressions were compared with the Fisher's exact test for different subgroups. The Friedman test was used to test the difference of *H. pylori *density between the antrum, corpus and cardia of the stomach in each mouse. All tests were assessed two-tailed and a *p *value < 0.05 was taken as significant.

## Results

### The rates of successful *H. pylori *colonization in mice with different acquisition ages

On the 2^nd ^week after *H. pylori *challenge, *H. pylori *colonization was successful in 89% (8/9) of the young mice group and 100% (10/10) of the adult mice group. In contrast, on the 8^th ^week after *H. pylori *challenge, the colonization rate was significantly lower in the young mice than that in the adult mice (53% vs. 95%, *P *= 0.003). For those mice with successful colonization either on the 2^nd ^or the 8^th ^week, the densities of *H. pylori *were evenly distributed on the antrum, corpus, and cardia in both young and adult mice (*P *> 0.05).

### Age-specific gastric Lewis antigen and cytokines expressions in the naïve mice

**Figure 2:**
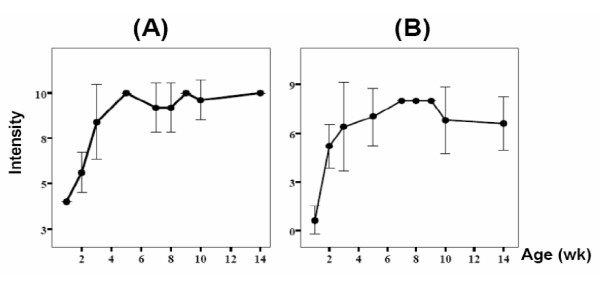
**The intensity of gastric Lewis antigens in naïve mice at different ages of life (A) Lewis x, (B) sialyl-Lewis x**.

In the naïve mice of both young and adult groups, the expressions of Le^x ^and sialyl-Le^x ^were weak on the gastric antrum. However, a gradual increase of the total intensity of Le^x ^and sialyl-Le^x ^expressions was disclosed in the age increments (Figure 2A &2B). Such an increase reached a plateau from the chronological age of near 5 weeks old. At the 2^nd ^week and the 8^th ^week after *H. pylori *challenge, the Le^x ^expressions were not different between the colonized and the control mice in both young and adult groups.

**Figure 3:**
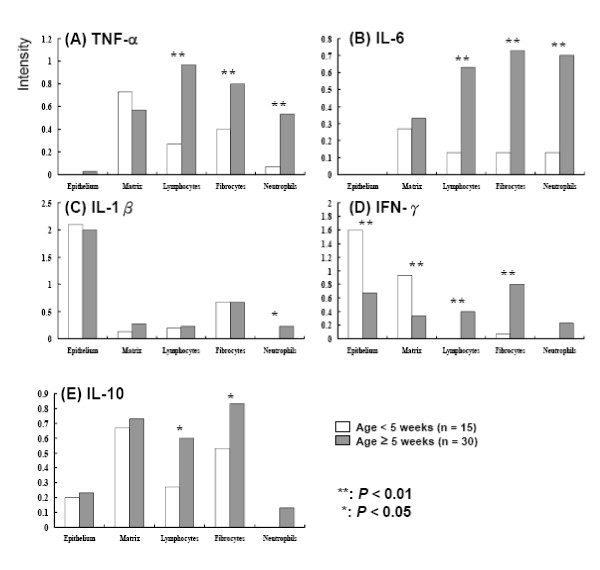
**Gastric cytokine expressions on the epithelium and lamina propria between naïve mice aged ≥ 5 weeks and < 5 weeks**. (A) Tumor necrotic factor alpha (TNF-α), (B) interleukin 6 (IL-6), (C) interleukin 1 beta (IL-1β), (D) interferon gamma (IFN-γ), and (E) interleukin 10 (IL-10).

The intensity of gastric cytokine expression varied between the different ages of mice and location. For the proinflammatory cytokines, there were no positive stainings of TNF-α and IL-6 on superficial epithelium in any of the naïve mice. The positive stainings of TNF-α and IL-6 on the lymphocytes, fibrocytes, and neutrophils over the lamina propria were significantly higher in mice aged ≥ 5 weeks than those aged < 5 weeks (*P *< 0.01; Figure 3A &3B). The positive staining of IL-1β was mainly found on the epithelium and expressed early in mice aged < 5 weeks. The rate of positive staining of IL-1β on neutrophils was higher in mice aged ≥ 5 weeks than those aged < 5 weeks (*P *< 0.05; Figure 3C).

In the early life of the mice, the positive IFN-γ staining was in the majority found on the epithelium and matrix, as the intensity of positive IFN-γ staining decreased after age ≥ 5 weeks (*P *< 0.01, Figure 3D). However, a significantly higher rate of positive IFN-γ staining on lymphocytes and fibrocytes was disclosed in the mice aged ≥ 5 weeks than those aged < 5 weeks (*P *< 0.01).

In contrast to the heavy IFN-γ staining on the epithelium in the early life of mice, IL-10 was usually negative on the epithelium. The positive staining of IL-10 was higher on lymphocytes and fibrocytes in the mice aged ≥ 5 weeks than in those aged < 5 weeks (*P *< 0.05; Figure 3E).

### Mice histology and Lewis antigen expression after *H. pylori *infection

On the 2^nd ^week after *H. pylori *challenge, the *H. pylori*-colonized young mice seemed to have a marginally higher rate of high TCIS (score ≥ 4) than the control mice (88% vs. 50%, *P *= 0.15), but such a marginal significance was not disclosed in the adult mice (30% vs. 30%, *P *= 1.00). Moreover, in the young mice group, the mice with persistent colonization on the 8^th ^week had an evidently higher rate of high TCIS than the mice that lost colonization (40% vs. 11%, *P *= 0.30). On the 2^nd ^week after *H. pylori *challenge, the percentage of colonized young mice having a high intensity of sialyl-Le^x ^expression (score ≥ 7) had dropped compared to that in the control mice (25% vs. 60%, *P *= 0.19). However, such a drop was not evident on the 8^th ^week (100% vs. 90%, *P *> 0.05) and with adult mice on the 2^nd ^week (90% vs. 100%, *P *> 0.05) and the 8^th ^week (58% vs. 60%, *P *> 0.05) after *H. pylori *challenges.

### Gastric cytokines expression after *H. pylori *infection

On the 2^nd ^week after *H. pylori *challenge, the young mice with successful colonization had a significantly higher intensity of IL-6 on fibrocytes (75% vs. 0%, *P *= 0.002) and matrix (75% vs. 20%, *P *= 0.05), but lower IFN-γ (0% vs. 100%, *P *< 0.001) on the matrix than the naïve controls (Table 2). Also in the adult mice, the mice with successful colonization were disclosed to have a lower rate of IFN-γ expression on fibrocytes than the control mice (30% vs. 90%, *P *= 0.02).

**Table 2:** The gastric cytokine expressions on the 2^nd ^week of young and adult mice with persistent *H. pylori *colonization and naïve control mice.

		Young mice			Adult mice		
		
Parameter (%)		Colonized (n = 8)	Control (n = 10)	*P *value	Colonized (n = 10)	Control (n = 10)	*P *Value
IL-6*	Matrix	75	20	0.05	70	30	NS
	Lymphocyte	50	10	NS	60	70	NS
	Fibrocyte	75	0	0.002	80	50	NS
	Neutrophil	37.5	20	NS	20	70	0.07

TNF-α *	Matrix	87.5	70	NS	80	90	NS
	Lymphocyte	62.5	40	NS	90	90	NS
	Fibrocyte	87.5	40	0.07	100	90	NS
	Neutrophil	12.5	10	NS	30	40	NS

IL-1β	Epithelium ≥ 2	100	70	NS	100	90	NS
	Matrix	^a^25	0	NS	100	80	NS
	Lymphocyte	25	20	NS	30	0	NS
	Fibrocyte	^a^25	60	NS	100	100	NS

IFN-γ	Epithelium ≥ 2	0	30	NS	10	0	NS
	Matrix	0	100	< 0.001	40	20	NS
	Lymphocyte	0	0	NS	10	30	NS
	Fibrocyte	12.5	10	NS	30	90	0.02
	Neutrophil	0	0	NS	10	20	NS

IL-10*	Matrix	^a^25	60	NS	100	80	NS
	Lymphocyte	25	20	NS	60	60	NS
	Fibrocyte	^a^37.5	50	NS	100	80	NS
	Neutrophil	12.5	0	NS	10	20	NS

CIS: chronic inflammatory score; AIS: acute inflammatory score. *: colonized means mice infected with *H. pylori *with successful colonization (positive urea test and histology) on sacrifice. ^a^: *P *value < 0.01; ^b^: *P *value ≤ 0.05 between the young and adult colonized mice. NS: no significance. *indicated on this parameter, the epithelium is rather weak for such cytokine on immunohistochemistry staining.

On the 8^th ^week after *H. pylori *challenge, the rates of high TNF-α (100% vs. 20%, *P *= 0.02) on the matrix and high IL-1β (100% vs. 20%, *P *= 0.02) on fibrocytes were higher in the young mice with persistent colonization than in the control mice (Table 3). For the adult mice with successful colonization, the rates of high IFN-γ (30% vs. 100%, *P *= 0.003), IL-10 (20% vs. 100%, *P *= 0.001) and IL-6 (50% vs. 100%, *P *= 0.03) on fibrocytes were also significantly lower than those of the control naïve mice. Nevertheless, it was more common in the colonized adult mice than the control mice to have a higher IFN-γ (80% vs. 40%), IL-10 (80% vs. 40%), and IL-6 (80% vs. 30%) on the matrix.

**Table 3:** The cytokine expressions on the 8^th ^week of young and adult mice with persistent and lost *H. pylori *colonization and naïve control mice.

		Young mice				Adult mice		
		
Variables (%)		Persistent colonization (n = 5)	Lost colonization (n = 4)	Control (n = 10)	*P*^† ^value	Persistent colonization (n = 10)	Control (n = 10)	*P *value
IL-6*	Matrix	100	80	50	NS	80	30	NS
	Lymphocyte	25	60	50	NS	70	70	NS
	Fibrocyte	100	80	70	NS	50	100	0.03
	Neutrophil	0	20	50	NS	20	70	NS

TNF-α *	Matrix	100	100	20	NS	100	60	NS
	Lymphocyte	100	80	60	NS	90	90	NS
	Fibrocyte	100	100	70	NS	100	80	NS
	Neutrophil	25	20	60	NS	10	40	NS

IL-1β	Epithelium ≥ 2	100	80	70	NS	100	80	NS
	Matrix	75	40	30	NS	100	80	NS
	Lymphocyte	50	60	0	NS	50	60	NS
	Fibrocyte	100	60	20	NS	100	100	NS
	Neutrophil	0	0	20	NS	0	10	NS

IFN-γ	Epithelium ≥ 2	0	80	20	0.048	30	0	NS
	Matrix	75	100	40	NS	80	40	NS
	Lymphocyte	75	60	40	NS	30	50	NS
	Fibrocyte	75	100	50	NS	30	100	0.003
	Neutrophil	0	0	40	NS	20	0	NS

IL-10*	Matrix	100	20	70	0.048	80	40	NS
	Lymphocyte	100	20	70	0.048	40	50	NS
	Fibrocyte	100	60	70	NS	20	100	NS
	Neutrophil	50	0	10	NS	0	10	NS

^†^: the significance was compared between young mice with persistent and lost colonization. *indicated on this parameter, the epithelium is rather weak for such cytokine on immunohistochemistry staining.

### Higher sialyl-Le^x ^coexists with higher IL-10, but lower INF-γ for *H. pylori *colonization

On the 2^nd ^week, the colonized young mice had no significant difference in the intensity of sialyl-Le^x ^expression than the control mice (*P *> 0.05). However, on the 8^th ^week after *H. pylori *challenge, the young mice with persistent colonization had a higher rate of evident sialyl-Le^x ^expression (score ≥ 7) than those of the mice that lost colonization (100% vs. 56%, *P *= 0.03). In addition to showing a higher rate of sialyl-Le^x ^expression (score ≥ 7), all of the persistently *H. pylori*-colonized young mice significantly coexisted with lower IFN-γ and higher IL-10 expressions than those mice that had lost persistent colonization on the 8^th ^week (Table 4, 100% vs. 0%, *P *= 0.008).

**Table 4:** A low gastric IFN-γ (score ≤ 1) expression on the epithelium coexisting with high sialyl-Le^x ^(score ≥ 7) and IL-10 (on both matrix and lymphocytes) was correlated to persistent bacterial colonization in the early acquisition age of mice.

Cytokine expression (%)	Colonization of young mice		OR (95% CI)	*P *value*
			
	Persistent (n = 4)	Lost (n = 5)		
Low IFN-γ	100	20	0.2 (0.1–1.2)	0.048
High IL-10	100	0	-	0.008
Low IFN-γ and high sialyl-Le^x^	100	0	-	0.008
Low IFN-γ and high IL-10	100	0	-	0.008

OR: odd ratio; CI: confidence interval; *: Fisher's exact test

Table 5 shows the scoring of IFN-γ on the epithelium and IL-10 on the matrix and lymphocytes between mice with persistent and lost colonization at the 8^th ^week after *H. pylori *challenge. None of the young mice with persistent bacterial colonization had IFN-γ expression on the epithelium but had obvious IL-10 expression on the matrix and lymphocytes as compared to the mice without colonization.

**Table 5:** The score of IFN-γ on the epithelium and IL-10 on the matrix and lymphocytes at the 8^th ^week after *H. pylori *challenge in the young mice with and without persistent colonization.

			Score of IL-10	
			
Mice No.	Colonization	Score of IFN-γ on epithelium	on matrix	on lymphocyte
1	Yes	0	1	1
2	Yes	0	1	1
3	Yes	0	1	1
4	Yes	0	1	1
5	No	1	0	0
6	No	2	1	0
7	No	2	0	0
8	No	2	0	0
9	No	2	0	1

## Discussion

Acquisition of *H. pylori *mainly occurs in childhood and spontaneous elimination of infection usually occurs in young children [3-6,20]. In this study, the C57BL/6 adult mice had a favorable *H. pylori *colonization rate of isolates ranging to more than 80%, which is compatible with previous reports [13,14]. This high colonization rate in adult mice is also compatible to support the fact that late acquisition of *H. pylori *in adult human patients usually results in chronic colonization [20]. Moreover, as compared to the 2^nd ^week of young mice after *H. pylori *acquisition, the colonization rate in the 8^th ^week was 53%, which supports that spontaneous elimination of *H. pylori *really occurs during the early acquisition age of mice. So the present study should be highly original to introduce a mouse model designed to validate that there should be a loss of *H. pylori *colonization after an earlier acquisition of *H. pylori*, which closely mimics the clinical findings in young children.

Our previous study has shown that the intensity of gastric Le^x ^is positively related to the increment of age in humans [12]. However, for those without *H. pylori *infection, the expression of sialyl-Le^x ^on gastric epithelium is weak or lacking in dyspeptic children and adults [12]. Compatible with humans, the intensity of gastric sialyl-Le^x ^of mice is also positively correlated to the increment of age (Figure 2B). Such a finding in mice is also compatible to a rat study, which disclosed an age-related increment of glycosylation in liver tissues [21]. Increasing gastric sialyl-Le^x ^expression after *H. pylori *infection has been reported in humans and Rhesus monkeys [9-12]. However, in our mice study, a decreased intensity of gastric sialyl-Le^x^was disclosed in the *H. pylori*-infected young mice, but not in adult mice on the 2^nd ^week, as compared to the control mice. More strikingly, on the 8^th ^week after *H. pylori *acquisition, such a decrease of sialyl-Le^x ^disappeared in both the colonized young and adult mice, which had a fully sialylated gastric mucosa. This finding suggests that *H. pylori *challenge should be involved to disturb the age-related maturation curve of sialylation in the young mice. Moreover, it indirectly suggests that the changes of sialyl-Lewis antigen in young mice should be highly correlated to the persistence or loss of *H. pylori *colonization.

In this animal model, early *H. pylori *acquisition caused a higher CIS than in controls on the 2^nd ^week (87.5% vs. 50%, *P *= 0.15), but not on the 8^th ^week (40% vs. 30%, *P *= 0.36) after *H. pylori *challenge. Assuming that dense inflammation causes more severe clinical symptoms, this histology change could be correlated to our previous findings that *H. pylori *infection could cause short-term epigastric pain in children, although usually resolved at follow-up [22]. Concerning both clinical observation and in vivo mice model data, *H. pylori *acquisition in children usually presents as a self-limited sequel in inflammation.

After *H. pylori *infection, the different host responses of the gastric cytokines could possibly be related to different clinical outcomes [14,18,23,24]. In this study, the naïve mice were found to have regular IL-1β and IFN-γ expressions (Figure 3C &3D) on the gastric epithelium, which is consistent with a previous report in humans [15]. Our mice study exhibited an age-specific cytokine expression on the gastric epithelium (IFN-γ) and lamina propria (IL-6, TNF-α, IFN-γ, and IL-10). Due to the age-specific response of the cytokines, our study supports that there should be different naïve immune responses between children and adults.

Based on the immunohistochemical study, this study confirmed that the pro-inflammatory cytokine expressions on the gastric lamina propria could change on both the 2^nd ^and the 8^th ^week after *H. pylori *challenge [25,26]. Both in mice and in humans, the gastric cytokine responses of *H. pylori *infection have been demonstrated to be Th1-dominant, because the level of IFN-γ is increased in *H. pylori*-infected subjects compared to controls [15,18,23,27]. A high INF-γ level has been attributed as hallmark of *H. pylori*-related gastritis, especially during chronic inflammation and potentially attributed as a protective role against *H. pylori *colonization [16-18,28]. As shown in Table 3, we disclosed that young mice with a loss of colonization had a higher IFN-γ expression on the epithelium than the mice with persistent colonization (*P *= 0.048). This finding is compatible with a human study in children which showed that children with *H. pylori *infection had a lower concentration of IFN-γ than in non-infected controls [29]. This data in mice suggests that there may be a significant correlation to colonization loss once there is a high expression of IFN-γ. Therefore, it should be concerned to survey the different host response of IFN-γ that can be related to have different *H. pylori *infection outcomes in children.

IL-10 is a Th2 cytokine, and can be triggered by compensatory regulation after a Th1 shift by the *H. pylori *challenge to protect the gastric mucosa [13,15,23,27]. Therefore, it is not so striking to illustrate a significant increase of IL-10 expression in the young mice with persistent colonization (Table 3). As shown in Table 4, the young mice with low IFN-γ on the epithelium to have a persistence of *H. pylori *colonization had a 100% coexistence with a high IL-10 expression on the matrix and lymphocytes. These data once again support that upregulation of IL-10 in such mice should be a co-existed compensatory regulation after a drop in IFN-γ to serve as a protective role for the infected mucosa. In addition to coexistence with high IL-10, the persistently *H. pylori*-colonized young mice exclusively had low IFN-γ and high sialyl-Le^x ^co-expressions on the epithelium compared to those without colonization. These data once again suggest that the host response having a low IFN-γ should somewhat coexist with a high sialylation of Lewis antigen, which can possibly serve as putative receptors for *H. pylori *colonization.

## Conclusion

In conclusion, this animal model with early acquisition of *H. pylori *represents a similar course of *H. pylori *infection to that in early childhood. A high expression of sialyl-Le^x ^and a depletion of IFN-γ on the gastric epithelium coexisting with high IL-10 in infected young mice should be related to maintaining the persistent colonization of *H. pylori*. Targeting the sialylation or enhancing the IFN-γ expression on the gastric epithelium should be promising to decrease the persistent colonization of *H. pylori *in childhood or even in adulthood.

## Competing interests

The authors guarantee that there is no financial relationship with any company. There are no competing interests including the study design, the collection, analysis, and interpretation of data, the writing of the report, and the decision to submit the paper for publication.

## Authors' contributions

YYJ contributed to the study design, study performance, and wrote the first draft of the manuscript. YHB, an experienced pathologist, read the histology and Lewis staining of each biopsy. WJJ also contributed to the study design, technique consultation, and paper revision. SBS, the correspondence author, organized the whole study design, team discussion, and final revision of this paper.
